# Self-healing dental biomaterials: bioinspired pathways to sustainable dentistry

**DOI:** 10.2340/biid.v12.45229

**Published:** 2025-12-29

**Authors:** Aftab Ahmed Khan

**Affiliations:** Dental Health Department, College of Applied Medical Sciences, King Saud University, Riyadh, Saudi Arabia

**Keywords:** self-healing, sustainable biomaterial, resin composite, adhesive, resin cement, biomimicry, dynamic covalent network

## Abstract

**Objective:**

Resin composite restorations typically last 6–10 years but often fail due to mechanical fatigue, hydrolytic breakdown, and degradation at the interface. These failures result in frequent replacements, leading to significant clinical and environmental impacts. Extending restoration durability is essential for both patient care and sustainability. This review examines recent advances in self-healing dental biomaterials, emphasising the underlying chemical and physical mechanisms, their integration into resin composites, cements, and adhesives, and their relevance to sustainable restorative practice.

**Materials and methods:**

A narrative review methodology was employed to synthesise the current evidence. Studies on self-healing mechanisms, including extrinsic (capsule- or reservoir-based) and intrinsic (dynamic covalent, supramolecular, or shape-memory polymer) systems, were critically evaluated with emphasis on evidence relevant to dental resin composites, cements, and adhesives. Parallel insights from polymer and material sciences were included where dental-specific research was limited. Supplementary searches were conducted on Google Scholar for additional peer-reviewed articles, books, and preprints.

**Results:**

Experimental resin composites and cements incorporating microcapsule-based self-healing systems exhibited fracture toughness recovery between 65% and 77%, maintaining structural integrity after 6 months of water storage in deionised water at 37°C. Disulfide and Diels–Alder dynamic networks, though mostly investigated in polymer science, show potential for repeatable healing under mild triggers, while supramolecular hydrogen-bonding and bioactive fillers offer adaptive repair and remineralisation at adhesive interfaces. Self-healing strategies align with the four pillars of green dentistry, that is pollution prevention, water conservation, energy efficiency, and waste reduction by potentially halving procedural resource consumption through extended restoration lifespan.

**Conclusion:**

Self-healing biomaterials designed and developed in accordance with sustainability principles have the potential to transform restorative dentistry by facilitating autonomous repair, prolonging restoration lifespan, and minimising the environmental footprint through reduced material usage and clinical waste generation.

CLINICAL SIGNIFICANCE

Sustainability-driven self‑healing dental biomaterials represent a paradigm shift in restorative dentistry by enabling restorations to autonomously repair microcracks, adapt to the harsh oral environment, and maintain mechanical integrity over time. By prolonging the lifespan of dental resin composites, cements, and adhesives, these materials reduce restoration replacement frequency, preserve tooth structure, and enhance long-term clinical outcomes while minimising patient discomfort and costs. Beyond patient-level benefits, their durability also supports environmentally sustainable practice by minimising procedural waste, energy, and material consumption, positioning self-healing systems as a clinically and ecologically meaningful advancement in future dental care.

## Introduction

### The clinical problem: failure of dental restorations

Although the global prevalence of dental caries has declined in recent decades, untreated caries in permanent dentition remains widespread, affecting ~35% of the population [1]. The direct treatment of dental diseases imposes an estimated annual cost of US$298 billion, accounting for about 4.6% of global health expenditure [1, 2].

Dental resin composites represent the preferred material for direct aesthetic restorations [3–5], with millions of restorations performed annually worldwide. In the United States (US) alone, it is estimated that ~200 million dental restorations are placed each year [6, 7], underscoring the high demand worldwide. Despite their popularity, their average clinical lifespan is only 6–10 years [7, 8]. The failure of dental resin composite can be influenced by factors such as secondary caries, bruxism, cavity size, and operator technique [1, 9]. However, primary failure causes relate to the material’s properties, including breakdown of the resin matrix and the interface between filler and resin matrix, exacerbated by mechanical fatigue, chemical degradation, and cumulative oral environmental challenges [10–12]. These failure modes necessitate replacement restorations, which tend to be more extensive than the originals [13], initiating a destructive cycle that compromises tooth structure and increases patient discomfort and treatment costs [5].

### The environmental imperative: the need for sustainable dentistry

The dental industry exerts a significant environmental impact throughout the life cycle of dental restorations. This includes energy-intensive production of materials such as monomers and fillers, the extensive use of single-use plastics in barriers, instruments, and packaging during treatments, and the disposal of failed restorations, which generates significant plastic and hazardous waste [14, 15]. In addition, dental practices consume substantial amounts of water and energy, further contributing to their ecological footprint [16]. Sustainable dentistry, or ‘green dentistry’, aims to reduce this environmental burden through waste reduction, pollution prevention, and conservation of resources [16]. Central to this approach is enhancing the durability and lifespan of dental materials to reduce replacement frequency and associated waste production.

### The paradigm shift: learning from nature

Living organisms possess remarkably sophisticated self-repair mechanisms that ensure survival and functional continuity in hostile and changing environments. These mechanisms are rarely wasteful; instead, they are adaptive, resilient, and energy-efficient, enabling organisms to restore function without the need for complete replacement of damaged structures. This principle contrasts sharply with current restorative dental practice, where materials are largely passive, prone to cumulative damage, and often require full replacement once failure occurs.

The scientific discipline of biomimicry seeks to translate these biological strategies into technological and material innovations. Rather than designing materials that are inert and prone to failure, biomimetic approaches aim to engineer systems that can sense damage, respond adaptively, and restore function autonomously. In dentistry, this paradigm shift moves from inert conventional materials towards dynamic, sustainable self-healing biomaterials inspired by natural healing processes.

Sustainable self-healing dental materials offer not only extended restoration lifespan and reduced replacement frequency but also align with sustainability and environmental principles [17]. By integrating repair, mechanisms inspired by nature, such as controlled release of remineralising agents, microcapsule-based healing systems, or bio-derived polymers, these materials offer a dual advantage, that is clinical superiority through enhanced durability and environmental responsibility through reduced material wastage and energy use. This new class of biomaterials embodies a holistic approach that integrates oral healthcare with the resilience, adaptability, and sustainability characteristic of natural environments.

This review aims to comprehensively synthesise recent advancements in sustainable self-healing strategies for dental biomaterials. It explores the biological principles underlying self-healing, the chemical and physical mechanisms involved, and the application of these technologies across various dental material classes. Central to the discussion is a critical evaluation of these innovations in enhancing material longevity, thereby promoting sustainable dentistry. The review also addresses key challenges to clinical translation and propose future research directions for the field.

### Literature search strategy

The review employed a narrative review approach. Comprehensive searches were performed in PubMed, Scopus, and Web of Science using key terms such as ‘self-healing’, ‘dental composite’, ‘dental adhesive’, ‘biomimicry’, and ‘sustainability’. Parallel insights from polymer and material sciences were included where dental-specific research was limited. Supplementary searches were performed on Google Scholar for additional peer-reviewed articles, books, and preprints.

## Biomimicry: natural models and material design principles

Biological systems possess remarkable self-repair mechanisms that ensure resilience and longevity [18–20], providing valuable templates for the development of next-generation dental biomaterials. For instance, the dentin–pulp complex exhibits a regenerative mechanism when subjected to mild carious attack [21]. Odontoblasts and progenitor cells within the pulp are capable of secreting reactionary dentin, a process orchestrated by signalling molecules such as transforming growth factor-β and bone morphogenetic proteins, which act as biochemical signals for repair and mineralisation [22, 23]. This natural defence mechanism highlights the potential of bioinspired restorative materials that can stimulate or mimic similar regenerative pathways.

Bone tissue offers another sophisticated model. It is a dynamic, hierarchically organised composite that undergoes continuous remodelling through the coordinated activity of osteoclasts and osteoblasts [24, 25], thereby ensuring both mechanical competence and mineral homeostasis. Microdamage incurred during routine mechanical loading is repaired via targeted remodelling, supported by the release of local growth factors and signalling cascades [26, 27]. This dynamic equilibrium provides a direct analogy for self-sustaining material systems that maintain structural integrity through an intrinsic, continuous supply of healing agents. Human skin, too demonstrates an extraordinary capacity to close wounds through a coordinated cascade of haemostasis, inflammation, proliferation, and remodelling, ultimately regenerating functional tissue [28].

In the plant kingdom, wound sealing represents a robust ecological strategy. When injured, many trees initiate a process of compartmentalisation of decay in trees by forming lignified barriers and exuding resins or latex [29]. These hydrophobic exudates harden upon exposure to air, effectively sealing the wound, reducing moisture loss, and preventing microbial invasion [30]. This concept has directly inspired extrinsic microcapsule-based healing approaches, where encapsulated monomers or adhesives are released upon crack propagation to reseal damaged areas.

Spider silk is a natural material known for its outstanding strength and toughness, which originates from its special internal structure [31]. The silk contains small β-sheet crystals surrounded by softer protein chains; when the silk is stressed, hydrogen bonds within this structure can break and regroup, helping absorb force and keep the silk stable [32]. This mechanism parallels the design of intrinsic self-healing polymers, which rely on reversible non-covalent interactions or dynamic covalent chemistry (DCC) to restore integrity after damage.

Beyond these widely cited models, several other biological systems provide further inspiration. Mussels, for example, adhere strongly to wet and mineralised surfaces by secreting catechol-rich proteins that form robust, reversible bonds, a strategy that has already guided the development of durable dental adhesives [33]. Similarly, nacre (mother-of-pearl) found in mollusc shells has a brick-and-mortar-like structure, where tiny mineral plates are held together by natural polymers [34]. This special design makes it extremely tough by stopping cracks from spreading and absorbing energy. Modern hybrid ceramics are designed using the same principle. Regeneration in starfish and amphibians offers another paradigm: through cellular dedifferentiation, proliferation, and re-patterning, these organisms can restore complex tissues [35], inspiring bioactive materials that might 1 day direct pulp or periodontal tissue regeneration. Finally, marine mineralisers such as oysters and corals orchestrate the deposition of highly ordered, hierarchically structured calcium carbonate skeletons, serving as models for controlled biomineralisation in synthetic restorative systems [36, 37].

Natural systems exhibit diverse healing strategies such as cell proliferation, layered construction, continuous repair, and effective adhesion. These concepts guide the design of restorative dental biomaterials that mimic nature’s reparative functions. It is important to distinguish biomimicry, which seeks to closely replicate natural processes, from bioinspiration, which adapts key natural ideas into novel synthetic designs [38]. Incorporating microcapsules that release healing agents upon crack formation exemplifies bioinspiration in dental materials. This strategy replicates nature’s repair function without duplicating exact biological structures. The focus lies on mimicking function – autonomous repair – rather than precise form, allowing material design to leverage nature’s evolutionary optimisation for effective, adaptable, and sustainable dental biomaterials.

## Fundamental mechanisms of self-healing

Self-healing mechanisms can be broadly classified into two categories: extrinsic and intrinsic. Understanding these mechanisms provides the foundation for designing dental materials capable of autonomous repair.

### Extrinsic (autonomous) healing

Extrinsic systems incorporate a discrete healing agent embedded within the material matrix. Damage ruptures these capsules, releasing agents that polymerise or react to seal cracks autonomously, without external intervention [39]. Common designs incorporate microcapsules (10–300 µm) with polymeric shells containing liquid healing monomers (e.g. dicyclopentadiene) and dispersed catalysts in the matrix, enabling ring-opening metathesis polymerisation (ROMP) upon crack formation [40]. The catalyst for polymerisation (e.g. Grubbs’ catalyst) is dispersed within the surrounding matrix. Upon crack-induced rupture, the monomer is released, comes into contact with the catalyst, and undergoes ROMP, solidifying and healing the crack [41, 42]. In dentistry, biocompatible monomers such as triethylene glycol dimethacrylate (TEGDMA) and catalysts are being explored.

Other extrinsic approaches aim to provide autonomous healing in dental materials while remaining practical for clinical applications. One example is the use of refillable micro-reservoirs or embedded reactive fillers, which release healing agents upon damage [43]. These systems mimic nature’s strategy of storing and delivering repair agents without requiring a full replication of complex circulatory-like networks. While the idea of large-scale vascular-inspired systems has been explored in structural and engineering materials, their direct translation into dental restoratives is not feasible due to the small scale of restorations, mechanical property requirements, and strict biocompatibility constraints. Therefore, contemporary dental research focuses on compact, refillable, or responsive reservoirs that can deliver healing agents effectively within the thin layers of dental resin composites, cements, or adhesives.

### Intrinsic (non-autonomous) healing

Intrinsic self-healing materials do not rely on a separate healing agent. Instead, the material itself is designed with an inherent ability to heal damage through reversible physical or chemical interactions [44]. These often require an external stimulus such as heat, light, or pressure to initiate the healing process [45].

#### Reversible covalent bonds

Reversible covalent chemistry is a promising intrinsic self-healing approach because it allows polymers to undergo multiple repair cycles within their own network [46]. Unlike microcapsules that release a limited amount of healing material once, reversible covalent bonds can break and re-form multiple times, facilitating repeated repairs throughout the material’s life [47]. Two main strategies have gained attention in dental materials research:

The reversible covalent reaction between furan and maleimide groups, known as the Diels–Alder and reverse Diels–Alder reactions, has been adapted in experimental dimethacrylate-based polymer systems for dentistry [48]. The Diels–Alder reaction is a thermally reversible chemical process where a diene and a dienophile form a stable ring structure through covalent bonding. When moderate heat (around 80–120°C) is applied, this bond can dissociate again via the reverse Diels–Alder reaction, enabling the temporary softening and re-networking of the polymer, and heal cracks by reforming covalent crosslinks [49]. This reversible bonding mechanism allows damaged polymer chains to reconnect, giving the material the ability to restore its structural integrity multiple times [50]. Although body temperatures are lower, this principle provides a blueprint for engineering materials that can heal during controlled external stimulation (e.g. chairside heat application or light-emitting diode (LED)-assisted heating).

Another relevant mechanism is disulfide linkages, inspired by natural proteins such as keratin, where these bonds provide both strength and flexibility [51]. In dental resin, methacrylate monomers containing disulfide groups have been incorporated to enable autonomous crack healing [48]. When stress or mild stimuli (light, heat, or even mechanical friction) are applied, the disulfide bonds undergo exchange reactions, which reconnect the broken polymer chains and repair microcracks [52]. Studies have shown notable recovery of scratch resistance and partial restoration of strength in such systems [53, 54].

Compared to capsule-based approaches, reversible covalent systems enable repeatable healing at the molecular level and offer improved reliability, though they require thermal activation often exceeding physiological limits and still face challenges balancing mechanical strength with healing efficiency [55]. Their long-term stability under moist, enzymatically active oral conditions also warrants further investigation.

Beyond reversible covalent mechanisms, intrinsic self-healing can also be achieved through supramolecular chemistry-based self-healing systems, which rely on dynamic and reversible non-covalent interactions, enabling repair without the need for permanent chemical changes [56]. Key mechanisms include hydrogen bonding, where multiple directional bonds, such as ureidopyrimidinone motifs, can dissociate and reassociate, allowing materials to soften upon heating and regain structure on cooling [57]; ionic interactions, in which polymers with ionic side groups form reversible ionic clusters that act as cross-links, promoting healing under thermal or mechanical stimuli [58]; and host–guest interactions, where macrocyclic molecules like cucurbiturils or cyclodextrins form selective and reversible complexes with guest molecules, thereby introducing dynamic cross-links into polymer networks [59]. Collectively, these supramolecular strategies provide tunable and repeatable healing capabilities, though their mechanical robustness under long-term intraoral conditions remains an area of active research.

A further intrinsic approach uses shape-memory polymer transitions to restore structural continuity by physically closing micro-gaps. These polymers can ‘remember’ their original shape and return to it after being deformed when an external stimulus (usually heat) is applied [60]. In a self-healing context, shape memory polymer can be programmed to a permanent, crack-free state. When a crack occurs, heating the material above its transition temperature causes it to recover its original shape, closing the crack. Healing can be further enhanced if the polymer also contains reversible bonds that form new connections across the closed crack interface. A concise comparison of extrinsic and intrinsic self-healing mechanisms is provided in Table 1.

**Table 1:** Comparison of extrinsic and intrinsic self-healing mechanisms.

Feature	Extrinsic healing	Intrinsic healing
Healing agent	Encapsulated monomer + catalyst	The polymer matrix itself
Healing trigger	Crack propagation (autonomous)	External stimulus (heat, light, pH, etc.)
Number of healing events	Single (microcapsules), Multiple (vascular)	Multiple (theoretically unlimited)
Impact on mechanics	Can create stress concentrators	Generally minimal
Manufacturing complexity	Moderate to high	Low to moderate

## Self-healing strategies in key dental biomaterials

### Resin composites

**Figure 1:**
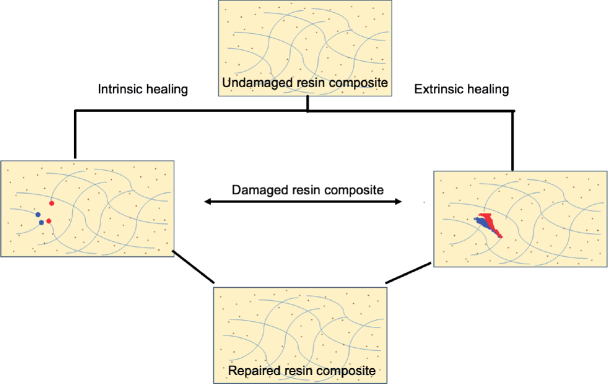
Schematic of self-healing in resin composites, showing intrinsic (matrix-based) and extrinsic (capsule-based) mechanisms leading to a repaired material.

Resin composites remain the most investigated platform for self-healing strategies, primarily due to their widespread use in restorative dentistry and their vulnerability to polymerisation shrinkage, hydrolysis, and fatigue-driven microcrack formation. The integration of healing mechanisms into this class of materials has been explored using both extrinsic and intrinsic approaches. These strategies are schematically illustrated in Figure 1.

Early work incorporating *TEGDMA–DHEPT-filled poly(urea-formaldehyde) (PUF) microcapsules* demonstrated self-healing behaviour in experimental resin composites. At capsule loadings of 10–20 wt.%, these materials achieved ~65% recovery of fracture toughness following crack propagation, with no major cytotoxicity detected *in vitro*, suggesting acceptable biocompatibility [61]. In a separate dose–response investigation, similar systems achieved up to ~75% recovery at ~10 wt.% capsule loading, confirming the efficiency of such extrinsic designs while emphasising the importance of optimising filler content to avoid compromising baseline strength [62].

More recently, the American Dental Association described a prototype ‘healing powder + encapsulated liquid’ composite, where alumina/silica or strontium fluoroaluminosilicate particles are paired with water/polyacrylic acid contained in silica nanocapsules. Upon microcrack formation, the powder and liquid react to form a resealing phase. This concept represents a practical, materials-transfer approach distinct from ROMP-based systems, with promise for clinical scalability [63].

While most intrinsic self-healing research currently resides in polymer science, there is growing evidence that disulfide-exchange chemistry holds promise for dentistry. For example, a waterborne polyurethane system featuring 4-aminophenyl disulfide exhibited dynamic bond reshuffling even at room temperature, with the exchange reaction catalysed by common oral stimuli such as pH changes or mild heat. This mechanism suggests potential for repeated, autonomous crack repair in resin-based dental systems without external healing agents [64]. Although not yet applied directly to resin composites, similar thiourethane-modified resin composites have demonstrated significantly enhanced toughness and lower polymerisation stress – properties indicative of beneficial sulphur-bond dynamics [64].

**Figure 2:**
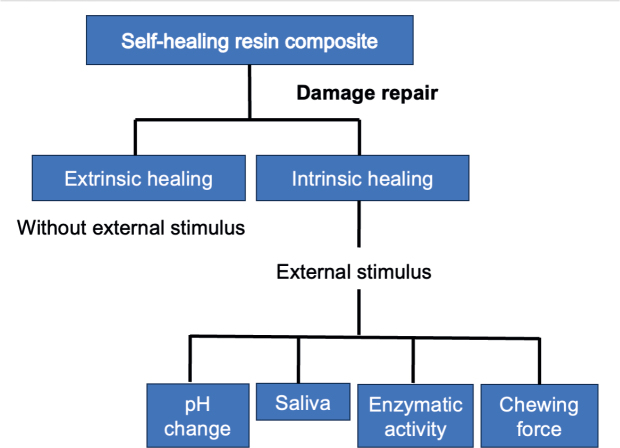
Damage repair mechanisms in self-healing resin composites Extrinsic and intrinsic healing pathways are shown, with intrinsic healing triggered by external stimuli such as pH changes, saliva, enzymatic activity, and chewing forces.

Diels–Alder and reverse Diels–Alder chemistries have been studied extensively within the polymer community for self-healing coatings and composites. These systems typically achieve 60–80% recovery of mechanical integrity upon heating (e.g. ~80% scratch healing on glass substrates) [65]. While these studies have been performed in non-dental systems such as coating films, the underlying chemistry – a thermally reversible bond capable of multiple healing cycles – provides a strong conceptual foundation for adaptation to dental adhesives or resin composites. These extrinsic and intrinsic repair pathways, and their dependence on physiological stimuli, are illustrated in Figure 2.

Although self-healing antifouling coatings based on supramolecular chemistries have not yet been applied directly as restoratives, they offer promising analogues. Dynamic non-covalent interactions at the surface interface, such as between a restoration and tooth, could potentially enable adaptive repair.

### Resin cements and adhesive systems

#### Capsule-based self-healing resin cements

Similar to resin composites, the concept of self-healing in resin cements is based on the intrinsic ability of the material to detect and repair microdefects at the tooth–cement interface. This repair occurs through the release of reactive agents or the activation of reversible chemical bonds, which restore interfacial integrity and maintain long-term adhesion without external intervention [66]. When a crack forms in the polymer matrix, the embedded microcapsules rupture and the healing liquid is released into the crack zone. The released liquid then encounters the catalyst dispersed in the polymer matrix, initiating polymerisation that fills and seals the crack [67]. A pioneering study developed experimental dental luting cements embedded with microcapsules containing a healing agent. Incorporation of 7.5% PUF microcapsules filled with TEGDMA enabled autonomous microcrack repair with 68–77% recovery of fracture toughness. The modified cement maintained dentin bond strength, mechanical integrity, and durable self-healing performance after 6 months of water ageing at 37°C [68].

#### Dynamic covalent and disulfide-based adhesive systems

Although self-healing dental adhesives employing DCC have not yet been reported, a recent study demonstrated the application of DCC at the resin–filler interface in dental restorative composites to facilitate stress relaxation and healing. Functionalised silica nanoparticles incorporated into bisphenol A glycidyl methacrylate (Bis-GMA)/TEGDMA resins induced a 30% reduction in polymerisation shrinkage stress and significantly enhanced mechanical properties, including a 60% increase in elastic modulus and a 33% improvement in flexural strength. Furthermore, the combination of DCC mechanisms within the resin matrix and at the interface resulted in an 80% reduction in shrinkage-induced stress. This strategy offers a promising avenue to improve composite performance and extend restoration longevity through persistent interfacial stress relaxation [69]. Complementing these findings, polymer-based studies have reported a disulfide-based thermoset adhesive exhibiting a fourfold increase in lap shear strength post-annealing compared to uncured systems and a twofold improvement relative to traditional networks, indicative of enhanced stress dissipation and adhesive durability [70]. Similarly, a bis-dynamic network integrating vinylogous urethane and disulfide bonds achieved ~87% healing efficiency under mild thermal conditions (100°C, 20 h), demonstrating reproducible repair in cross-linked matrices [71].

#### Supramolecular (hydrogen-bonding) adhesive systems

Conventional dental adhesives often suffer from polymer network heterogeneity, phase separation, and poor wet bonding, leading to rapid interfacial degradation. Although dental-specific studies remain limited, recent research has highlighted the potential of supramolecular-based adhesives developed from novel monomers containing multiple hydrogen-bonding motifs, particularly ureido-pyrimidinone (UPy) and catechol-functionalised structures, which were synthesised and incorporated into experimental formulations. These monomers promote dynamic and reversible bonding, forming strong supramolecular interactions with dentin collagen and hydroxyapatite. The resulting adhesives exhibit improved mechanical strength, enhanced moisture resistance, and superior interfacial stability compared with commercial controls [72]. Similar UPy- and catechol-based systems have also shown promise in broader biomedical applications, such as tissue engineering and wet-surface adhesion, demonstrating their versatility in biological environments [73, 74]. Translating this chemistry into dental adhesives could enable these materials to autonomously repair microdamage over time, significantly enhancing restoration longevity.

## The sustainability quotient: lifecycle perspective

While self-healing biomaterials are primarily developed to extend restoration longevity and reduce clinical failure, their broader value lies in how they align with the goals of sustainable dentistry. By decreasing the frequency of replacements, these materials directly reduce material consumption, clinical waste, and the ecological footprint of restorative dentistry.

## Integrating self-healing into the ‘green dentistry’ framework

**Figure 3:**
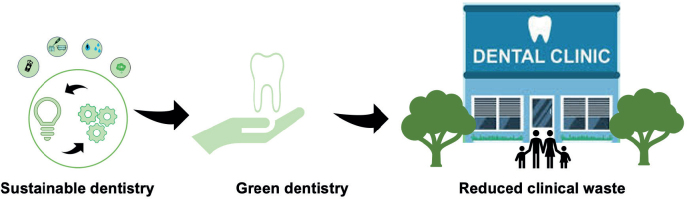
Conceptual overview of green dentistry highlighting key principles including prevention-focused care, material/resource efficiency, and environmentally sustainable clinical practices.

Sustainable dentistry has been described through four interlinked pillars: pollution prevention, water conservation, energy efficiency, and waste reduction [15, 75]. The last of these is particularly relevant to self-healing technologies, but all four are impacted. Pollution prevention is achieved as fewer replacements reduce the cumulative release of chemical waste and microplastic debris from discarded resin composites and adhesives [76]. Water and energy conservation result from avoided replacements, which save the sterilisation water and energy associated with autoclaving, lighting, and operating equipment [77]. Waste reduction occurs because extended restoration lifespan directly decreases the use of single-use disposables such as gloves, suction tips, and packaging, as well as restorative consumables such as composite aliquots, adhesives, etchants, and sharps including burs and carpules [78, 79]. Thus, self-healing resin composites and adhesives are not only restorative innovations but also key enablers of a greener clinical workflow. The integration of self-healing technologies into the green dentistry framework and their downstream public health impact are illustrated in Figure 3.

### Longevity versus manufacturing burden

The environmental impact stems not only from consumables, single-use plastics, and energy use but also from dental restorative materials (e.g. amalgam, composite, GIC), with amalgam and composite showing similar carbon footprints (~14.8 kg CO₂-eq) and GIC demonstrating somewhat lower carbon footprint [80]. A study estimated that ~800 million direct resin‑based composite (RBC) restorations were placed worldwide in 2015. With an average 10‑year failure rate, this corresponds to about 40 million replacements each year. Each replacement procedure generates significant particulate waste. Based on an average RBC waste weight of 0.3 g per restoration, clinical removal and replacement procedures were estimated to release roughly 12 tonnes of microparticle waste into municipal wastewater annually [81]. The analysis underscores that material longevity is the most critical determinant of overall environmental burden, as durable restorations that minimise replacement frequency substantially reduce life cycle impacts [15]. The study consequently advocates for prioritising durability in material selection, alongside adopting eco-friendly procurement, waste reduction, and enhanced recycling protocols to mitigate the ecological footprint of restorative dentistry [80].

However, the environmental performance of a restorative material cannot be assessed solely by its clinical longevity. A comprehensive evaluation must consider its entire life-cycle impact [80, 82]. Although restorative materials that involve complex manufacturing steps such as microcapsule incorporation, additional curing, or high‑energy processing may impose greater initial environmental costs, their extended service life can offset this burden. By reducing replacement frequency, such materials may ultimately lower total resource use, waste generation, and environmental impact over their operational lifespan.

A legitimate concern therefore is whether the advanced synthesis of self-healing systems (e.g. microcapsule encapsulation, DCC) carries a higher environmental footprint than conventional resin composites. Here, a life cycle assessment (LCA) framework is critical. Life cycle assessments quantify the cumulative energy demand, greenhouse gas emissions, and eco-toxicity of materials ‘from cradle to grave’.

Although dental-specific LCAs of self-healing systems are not yet reported, modelling from analogous self-healing products indicates a positive net effect, as extended service life offsets the additional manufacturing impacts [83, 84].

### Procedural waste, energy, and water savings

Dental practices are significant consumers of resources. Every replacement restoration generates consumables (e.g. gloves, barriers), restorative materials (composite, etchant, bonding), disposables (burs, anaesthetic carpules), and requires energy and water for lighting, suction, sterilisation, and handpiece irrigation [85, 86]. A comparative life cycle assessment in dental settings by Suresh et al. found that strategies aimed at extending restoration longevity, such as reducing replacement frequency, significantly lower environmental impacts. Also, the interventions such as using rainwater collection for sterilisation could reduce the per-patient carbon footprint by up to 90% for that component alone [87]. In another study, modelled environmental assessments by Duane et al. demonstrate that increasing restoration longevity substantially reduces the carbon footprint, waste, and energy use of dental care. Each avoided restoration replacement eliminates associated material, consumable, energy, and water consumption. Their findings indicate that doubling restoration lifespan can nearly halve environmental impacts, supporting material durability as a key lever for sustainable dentistry [16]. A recent observational study conducted across four dental practices and a United Kingdom (UK) dental teaching hospital found that dental care generates a substantial amount of single‑use plastic (SUP) waste. Among these, personal protective equipment (PPE) was identified as the main contributor. The study emphasised the urgent need for coordinated efforts by manufacturers, distributors, and oral healthcare providers to adopt reduction, recovery, and recycling strategies aimed at transitioning toward a circular plastics economy in dentistry [88]. Overall, enhancing material durability can halve resource consumption and emissions, underscoring its critical role in sustainable dentistry.

### Towards a circular economy in dentistry

**Figure 4:**
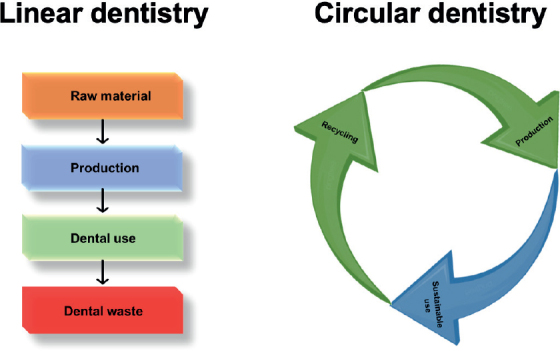
Comparison of linear and circular dentistry models highlighting waste generation versus sustainable use and recycling.

The linear ‘take-make-dispose’ model of traditional dental restorations results in continuous waste production and significant resource consumption throughout their lifecycle [80, 81]. A circular model instead emphasises durability, repair, and keeping materials in use for as long as possible [15, 80]. This paradigm shifts from linear to circular dentistry is depicted in Figure 4.

Self-healing dental materials represent a shift toward sustainability by autonomously repairing microdamage and delaying failure. This preserves the material’s function and reduces waste generation. This approach mirrors nature’s resilience and regeneration strategies, linking biological principles to sustainable dentistry. As described in Section 2, self-healing mechanisms align with the goals of a circular economy, aiming to separate clinical practice from continuous resource consumption. A hypothetical life cycle comparison of conventional and self-healing resin composites is presented in Table 2, highlighting potential reductions in carbon footprint, energy, water use, and hazardous waste.

**Table 2:** Environmental impact comparison: conventional versus self-healing composite (hypothetical LCA).

Impact category	Conventional resin composite (2 restorations over 15 years)	Self-healing resin composite (1 restoration over 15 years)	Net change
Global warming potential (kg CO^_2_^ eq)	X	Y	-50% or more
Cumulative energy demand (MJ)	A	B	-50% or more
Water consumption (Liters)	C	D	-50% or more
Hazardous waste generated (kg)	E	F	-50% or more

LCA: life cycle assessment.

X, Y, A, B, C, D, E, and F are hypothetical values used only for illustrative purposes, not actual measurements.

## Current challenges and limitations

### Biocompatibility of healing agents and byproducts

Ensuring biocompatibility is one of the most critical challenges in translating self-healing technologies to clinical dentistry. Many healing chemistries adapted from engineering materials, such as dicyclopentadiene-based systems, Grubbs’ catalysts, and urea-formaldehyde microcapsule shells, exhibit cytotoxicity or elicit unfavourable biological responses, rendering them unsuitable for intraoral use [61, 83, 89]. The complex and sensitive environment of the oral cavity demands that healing agents and their byproducts do not compromise the health of surrounding tissues, including dental pulp cells, gingiva, and oral mucosa [90–92]. Consequently, the primary research imperative is the development of fully biocompatible, non-cytotoxic, and regulatory-approved self-healing systems that seamlessly integrate with existing dental materials.

Therefore, healing systems should preferentially utilise monomers that are chemically related to or derivatives of established dental monomers such as Bis-GMA, urethane dimethacrylate (UDMA), and TEGDMA, which have demonstrated longstanding clinical safety profiles [93, 94]. This approach facilitates regulatory approval while minimising unexpected toxicological effects. In addition, the design of polymerisation catalysts must prioritise high efficiency under mild conditions, while simultaneously eliminating or minimising cytotoxicity. The chemical reactions underlying self-healing must avoid generating leachable or volatile byproducts with potential toxicity or allergenicity [93, 94]. Comprehensive characterisation of reaction products and long-term biocompatibility testing are essential to ensure safety in the dynamic oral environment.

### Healing efficiency and repeated repair capability

Healing efficiency is quantitatively measured as the percentage of recovered fracture toughness or strength. While high efficiencies (>80%) are reported in ideal lab conditions [62, 95], achieving this in the complex, wet, and biofouling oral environment is challenging. A recent study found that self-healing resin discs incorporating bacteria and nutrients achieved up to 85.8% crack healing under controlled laboratory conditions; however, healing efficiency in clinical environments may drop below 50.0% due to the complex oral conditions [96]. Furthermore, for intrinsic systems, the healing efficiency can decrease with each cycle [97, 98]. Yahyazadehfar et al. evaluated self-healing dental composites with encapsulated healing agents and observed that while fracture toughness and fatigue life significantly improved after the first healing cycle, repeated cyclic loading caused gradual degradation of the healing efficiency. The microcapsules can debond or rupture unevenly, limiting repeated autonomous healing [97]. El Choufi et al. explained that intrinsic self-healing relies on reversible bonds within the polymer matrix, but over multiple damage-healing cycles, bond fatigue, incomplete reversibility, or competing side reactions can reduce healing capacity [99]. For extrinsic systems, refillable channel-based delivery concepts offer repeatable healing but remain technologically complex and currently impractical for dental restorations. Achieving clinically significant healing over multiple damage events remains a key hurdle.

### Triggering mechanisms in the oral environment

For intrinsic systems, the triggering mechanism must be practical and safe. Thermal triggering is common but applying heat at 60–90°C to a tooth is not clinically feasible due to the risk of pulpal damage [100]. Photo-triggering with specific wavelengths of light is a promising alternative, but light penetration to the depth of a crack within a resin composite is limited [100]. pH triggering or moisture-triggering are highly clinically relevant, as they are autonomous and directly linked to the cause of failure (caries) [96]. Developing efficient systems that respond to these biological stimuli is a critical research direction.

### Manufacturing complexity and economic viability

Scaling up the production of self-healing materials for widespread clinical use remains a significant challenge. Synthesising microcapsules with uniform size and mechanically stable shells at an industrial scale is both technically demanding and costly [101, 102]. Similarly, integrating refillable or channel-based healing concepts into restorative materials risks compromising their strength and handling properties [103]. Ultimately, the higher production cost of these advanced systems must be balanced against their extended service life. Developing robust economic models that demonstrate long-term savings for both clinicians and patients will be essential for successful adoption. These challenges, their clinical and environmental relevance, and potential strategies for overcoming them are summarised in Table 3.

**Table 3:** Key challenges in the development of self-healing dental materials, their clinical and environmental relevance, and proposed solutions to overcome these barriers.

Challenge	Why it matters	Proposed solutions
Biocompatibility	Healing agents/capsules may be cytotoxic	Use Bis-GMA/UDMA derivatives; non-toxic shell materials
Healing efficiency	Low recovery under wet oral environment	Optimise intrinsic chemistries, pH/moisture-responsive triggers
Repeated repair	Extrinsic systems only heal once	Develop refillable reservoirs; multi-mechanistic hybrids
Triggering mechanisms	Heat not feasible intraorally	Light, pH, or moisture-triggered systems
Manufacturing complexity	Difficult capsule fabrication and cost	Scale-up microencapsulation; adopt greener chemistries
Clinical translation	No standardised testing protocols	Adopt fatigue, thermocycling, biofilm challenge models

Bis-GMA: bisphenol A glycidyl methacrylate; UDMA: urethane dimethacrylate.

## Future perspectives and conclusions

### Multi-mechanistic and stimuli-responsive systems

The future generation of self-healing dental materials is anticipated to integrate multiple complementary mechanisms, rather than relying on a single healing strategy. Immediate autonomic repair can be facilitated through microcapsule-based systems capable of sealing microcracks upon initiation. In contrast, sustained or delayed healing may be achieved via intrinsic dynamic covalent networks, such as disulfide bond exchanges, which respond to external stimuli like light or mild thermal activation to restore more extensive structural defects. In parallel, bioactive fillers capable of releasing calcium and phosphate ions in response to acidic challenges can promote apatite deposition, sealing marginal gaps, and preventing secondary caries. Such synergistic, stimuli-responsive systems would provide a robust defence against the diverse failure modes encountered in the oral environment.

### The horizon of ‘living’ bio-hybrid materials

The cutting edge of research involves moving from bioinspired materials to engineered living materials. This could involve embedding engineered, non-pathogenic bacteria (e.g. *Bacillus subtilis* spores) within the material. These bacteria remain dormant until activated by moisture from a crack, at which point they metabolise a pre-embedded nutrient to produce a mineral (e.g. calcium carbonate) or a polymer that seals the crack. This represents the ultimate fusion of materials science and synthetic biology, creating a truly ‘living’ restoration.

### Roadmap for clinical translation and commercialisation

For self-healing systems to reach the clinic, translation must overcome the so-called ‘valley of death’ between laboratory proof-of-concept, and real-world application. This requires the establishment of standardised *in vitro* protocols that mimic clinical challenges such as fatigue loading, thermocycling, and biofilm exposure; comprehensive biocompatibility testing beyond cytotoxicity, including assessments of genotoxicity, immunogenicity, and microbiome interactions; and strong collaborations between academia and industry to address manufacturing scalability and regulatory hurdles (Food and Drug Administration (FDA), Conformité Européenne (CE) marking). Ultimately, carefully designed preclinical and clinical trials are needed to demonstrate not only self-healing efficacy but also statistically significant improvements in restoration longevity and cost-effectiveness compared with conventional materials.

## Conclusion: A synergistic future for clinical excellence and planetary health

The development of sustainable self-healing dental biomaterials represents a convergence of clinical need and environmental responsibility. By learning from nature’s strategies, these emerging ‘smart’ materials can overcome the limitations of traditional passive composites and can offer the ability to repair damage autonomously, extend restoration longevity, and reduce the frequency of retreatments. Such innovations can not only improve patient comfort and reduce treatment costs but also contribute to sustainability by lowering the material and energy demands of repeated procedures. Moreover, the integration of durability and self-repair into restorative systems can align oral healthcare with the broader principles of environmental stewardship and circular economy. Achieving this vision will require continued interdisciplinary collaboration among material scientists, chemists, biologists, clinicians, and environmental experts. While full clinical translation has not yet been realised, the ongoing progress indicates that next-generation dentistry can evolve into a paradigm that is more effective, preventive, and restorative – not only for patients but also for the planet.

## Data Availability

The data supporting this review are available from the corresponding author upon reasonable request.
